# Echocardiography-based left ventricular mass estimation. How should we define hypertrophy?

**DOI:** 10.1186/1476-7120-3-17

**Published:** 2005-06-17

**Authors:** Murilo Foppa, Bruce B Duncan, Luis EP Rohde

**Affiliations:** 1 Graduate Studies Program in Cardiology. School of Medicine. Federal University of Rio Grande do Sul. Porto Alegre – RS. Brazil

**Keywords:** cardiovascular disease, echocardiography, epidemiology, geometric patterns, hypertrophy, left ventricular, male, risk factors, ultrasonography

## Abstract

Left ventricular hypertrophy is an important risk factor in cardiovascular disease and echocardiography has been widely used for diagnosis. Although an adequate methodologic standardization exists currently, differences in measurement and interpreting data is present in most of the older clinical studies. Variability in border limits criteria, left ventricular mass formulas, body size indexing and other adjustments affects the comparability among these studies and may influence both the clinical and epidemiologic use of echocardiography in the investigation of the left ventricular structure. We are going to review the most common measures that have been employed in left ventricular hypertrophy evaluation in the light of some recent population based echocardiographic studies, intending to show that echocardiography will remain a relatively inexpensive and accurate tool diagnostic tool.

## Introduction

The diagnosis of left ventricular hypertrophy (LVH) has been incorporated in the clinical practice as an important marker of cardiovascular disease. Its prevalence depends on classification criteria and specific population characteristics, ranging from 3% in normotensive community-based samples [1] to about three-quarters of hypertensive patients [2]. Irrespective of other risk factors, those in the upper distribution of left ventricular mass have their risk at least duplicated for future cardiovascular morbidity and mortality, as summarized in one metanalysis [3].

Echocardiography has been clinically employed for more than 30 years, becoming one of the most important non-invasive imaging methods in the evaluation of cardiac morphology and dynamics. However, the apparent simplicity in LVH evaluation by echocardiography conceals several intrinsic and usually unrecognized critical steps that may limit its clinical validity.

This manuscript introduces the basic principles of left ventricular mass (LV mass) estimation by echocardiography, focusing on the potential limitations and discrepancies of such measurement, in order to provide the appropriate background for understanding the rather complex issue of defining the cut-off values for LVH diagnosis in populations. Although advances in echocardiographic techniques has minimized the impact of many of the methodological details discussed here, an understanding of these topics is important, since most of the clinical and epidemiologic studies currently published are based on the echocardiographic criteria here described. We also intend to give a critical evaluation of the diagnostic performance and clinical validation of this imaging method.

## Left Ventricular Measurement

Left ventricular mass is generally calculated as the difference between the epicardium delimited volume and the left ventricular chamber volume multiplied by an estimate of myocardial density. Following this principle, several methodologies have been used to calculate left ventricular mass and to define hypertrophy with its own flaws and strengths on each step (Table 1), resulting in a wide range of values. Probably, the most significant echocardiographic limitation is related to inadequate quality imaging. Population-based studies are not able to obtain complete imaging in almost a quarter of screened patients [4,5]. mainly due to inappropriate acoustic windows.

**Table 1:** Critical Steps in Determining and Interpreting Left Ventricular Hypertrophy using Echocardiography

1. Imaging – Mode and Acquisition
2. Estimating Left Ventricular Volume
3. Defining Border Limits – Conventions of Layer Measurements
4. Calculating Mass – LV Mass Formulas
5. Indexing for Body Size
6. Determining Cut-off Points
a. Using a reference sample (normality/statistical criteria)
b. Using prognostic data (driven by clinical endpoint)
7. Evaluation of Left Ventricular Structure
8. Role of Additional Factors in LVM Determination
9. Clinical Correlates Associated with LVH

### Imaging – Mode and Acquisition

Both M-mode and two-dimensional imaging can be employed to calculate left ventricular mass. M-mode imaging allows better endocardial border definition as it has greater resolution due to higher frame-rate, as long as adequate ultrasound beam positioning is ensured and ventricle shape approaches normality. Two-dimensional imaging, on the other hand, depicts the "real" ventricular shape and identifies regional motion abnormalities. However, the quality of two-dimensional imaging may be limited due to both lower lateral resolution and frame-rate. Additionally this option is more time consuming, limiting its use in epidemiological studies. Two-dimensional images are usually acquired both in paraesternal and apical views, depending on the geometrical formulas that are used.

Technological advances have joined both methods and partially minimized their limitations There are standardized and validated recommendations for the clinical use of two-dimensional determination of LV mass [6], However, two-dimensionally oriented M-mode, obtaining images perpendicularly from the longitudinal axis slightly above the papillary muscle level is widely employed in clinical practice and is accepted as an adequate alternative in epidemiological studies. Digital imaging has also made it possible to reconstruct diverse M-mode planes from two-dimensional images (so called anatomical M-mode), allowing better positioning, although the final image resolution is that of two-dimensional images. Although accurate [7], LV mass estimation using anatomical M-mode has not been adequately validated in clinical studies. Two-dimensional mode has also improved due to refined imaging processing technology, particularly second harmonic imaging. Also, built-in software for automatic border detection has been developed allowing calculation of real-time volumes. Although most of these newer technologies are widely available in commercially available ultrasound equipment, their potential for providing additional accuracy in the evaluation of LVH remains poorly characterized.

A relevant degree of variability in LV mass determination could be attributed to online measurement inaccuracies due to lower imaging resolution of older equipment, which could reach 10% of parietal thickness. Nowadays, variability due to on-line or off-line analysis of digitalized images calculations is of considerably smaller magnitude [8].

We will focus our discussion on M-mode estimation of LV mass, since most epidemiological reports use this imaging modality. Preference for M-mode is based on its technical feasibility and availability at the time when most studies were performed. However, despite adequate correlation with two-dimensional measurements [9], M-mode has been suggested to averagely underestimate LV mass in about 20 g [10]. Theoretically, two-dimensional imaging would be more adequate in samples of patients with cardiovascular disease, where LV shape assumptions play a critical role in LV mass estimation.

### Estimating Left Ventricular Volume

Determination of left ventricular volumes is accomplished using formulas that fit ventricular shape to primary geometric figures. Ellipse, cylinder, cone, and truncated polyhedrons have been employed and validated in normally shaped ventricles, although most studies have limited sample sizes [11]. Some geometrical assumptions are best fit using two-dimensional images while others can be performed assuming geometric forms from M-mode imaging.

If a two-dimensional approach is used, both area-length and truncated-ellipsoid models are feasible and reasonably accurate, with validated formulas [6]. Among other formulas for volume calculation, the modified Simpson's Discs Rule, which is greatly facilitated nowadays by built-in software, offers flexibility that allows accurate estimation of left ventricular volumes even in greatly distorted ventricles. Even though volumes determined by Simpson's Rule are frequently used for ventricular function determination, they are not routinely used to calculate LV mass, probably due to limitations resulting from poor epicardial delimitation in some patients. Other geometrical models have been proposed in the past [12,13], with variable use in clinical practice.

The method of cubed formulas, which incorporate only one dimension of the left ventricular cavity and assume an ellipsoid geometry, is the most widely used to calculate left ventricular volumes and mass in noninvasive laboratories worldwide. The broad acceptance of M-mode derived estimation relies on the fact that it was the first method that was validated and also on its technical simplicity. The one-dimensional approach, however, imposes more strict geometrical assumptions and amplifies the risk of inaccuracy, as measurement errors are cubed.

### Defining Border Limits – Conventions of Layer Measurements

**Figure 1:**
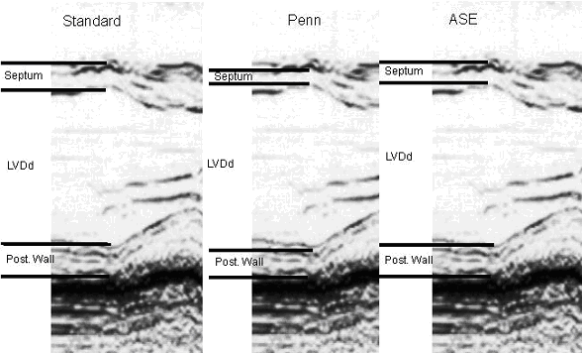
Comparison between M-mode border measurement conventions. The Standard convention measures from leading to trailing edge in the septum and from leading to leading edge of the posterior wall. Penn criteria excludes echoes from parietal walls while ASE criteria measure leading to leading edge. (LVDd: Left Ventricular Diameter in Diastole).

Ultrasound signals are reinforced where surfaces change density, allowing definition of limits between surface layers. The inclusion or exclusion of these echoes from interfaces of the left ventricular cavity or myocardial wall can cause significant discrepancies in the overall measurements [14]. Initial M-mode standard recommended inclusion of the edges as part of interventricular septum thickness, but exclusion of the posterior wall epicardial edge [15]. Investigators of the University of Pennsylvania developed a criteria (The Penn Convention) in which all edges are not included in parietal thickness measurements, but are considered as part of the ventricle cavity [16]. This approach underestimate LV mass when compared to the M-mode convention, proposed by the American Society of Echocardiography (ASE). This latter convention (ASE) is the most accepted border definition criteria, becoming the standard recommendation for M-mode estimations, and uses the leading edge of each layer [17](Figure 1). Employing Penn and ASE convention with the same volume formulas may originate LV mass discrepancies in the range of 15% in men and 18% in women [18]. Measurement convention must be acknowledged and adequately corrected for in comparisons of clinical studies of LVH.

In the past few years, spatial resolution of transthoracic echocardiography has greatly enhanced, leading to major improvements in image quality. Most of this progress can be attributed to second harmonic imaging, that significantly increases signal-to-noise ratio by receiving only harmonic frequencies. Although this technology is now widely available in most ultrasound equipment, its potential accuracy to evaluate LVH remains poorly characterized. A recent study suggests that LV mass estimations using second harmonic imaging can cause as much as a 26% increment in mean LV mass index corrected for body surface area, when compared to standard fundamental imaging using similar formulas and conventions [19]. This is probably due to an increase in border refringency.

### Calculating Mass (Left Ventricular Mass Formulas)

The most commonly used formulas to estimate LV mass are all variations of the same mathematical principle, based in the volume formulas stated above. Original calculations from Troy and coworkers were the first to be recommended as standard to estimate LV mass from M-mode measurements (Formula 1) [15].

Formula 1: LV mass(Troy) = 1.05 ([LVIDD + PWTD + IVSTD]^3^- [LVIDD]^3^) g.

Where: LVIDD = Left Ventricular Internal Diameter in Diastole

       PWTD = Posterior Wall Thickness in Diastole

       IVSTD = Interventricular Septum Thickness in Diastole

Subsequently, Devereux and colleagues suggested a slightly modified regression equation, using the Penn convention as the border definition criteria (Formula 2). Their prediction equation in this pivotal study was derived from necropsy findings of 34 patients [16].

Formula 2: LV mass(Penn) = 1.04 ([LVIDD + PWTD + IVSTD]^3^- [LVIDD]^3^) -13,6 g.

As depicted above, each regression equation was derived based on a specific border limits convention, an issue that is source of great confusion when interpreting different studies [20]. As expected, LV mass calculations derived from both formulas are linearly correlated, but final crude estimations may differ by more than 20%. Devereux and colleagues proposed a new adjusted equation, validated on necropsy findings of 52 individuals [21], using the ASE convention and accounting for this discrepancy (Formula 3).

Formula3: LVmass(ASE): 0.8 (1.04 ([LVIDD + PWTD + IVSTD]^3^- [LVIDD]^3^))+ 0,6 g.

Some critical aspects must be acknowledged regarding LV mass formulas. First, all necropsy validation studies have limited sample sizes and evaluate heterogeneous ventricular configurations. Second, these formulas may not perform adequately in distorted ventricles, where a two-dimensional approach is preferred. Different formulas may yield distinct cut point values, as demonstrated by Levy and coworkers in the Framingham cohort [18]. Finally, other post-mortem study showed only moderate correlation between echocardiographic and autopsy LV mass estimations (correlation coefficients ranging from 0.58 to 0.67) [22].

### Indexing for Body Size

Both body size and body habitus are clearly associated with LV dimensions and mass. Diverse normalization and indexes were created and tested to adjust for three different sources of physiologic variation in LV mass: lean body mass, obesity, and gender. However, the interdependence of such associations should be carefully understood to allow an adequate correction of LV mass without distorting its association with cardiovascular disease.

Several indexes for body size correction have been proposed, such as height, diverse allometric height adjustments, weight, body surface area, body mass index, and free-fat mass. The best way for normalization of LV mass is still controversial and another source of confusion. Different body-size adjustment criteria and their standard cut points result in different prevalence of patients with LVH [18]. Not surprisingly, those with higher LV mass are more frequently classified as hypertrophic by different classifications simultaneously [23].

The body surface area correction, using the Dubois formula [24], reduces variability due to body size and gender [25], but this index underestimates LV mass in the upper range of the body surface area distribution [26]. A correction based on height alone would allow evaluation of the separate role of obesity in LVH as proposed by Levy and coworkers [18].

Adjustment of LV mass with body surface area would imply that obese patients are expected to have higher LV mass estimations *per se*. In this scenario, height-based adjustments can more accurately estimate LV mass and the resulting cardiovascular risk associated with LVH in the obese. This turns to be particularly relevant in risk stratification due to the frequent clustering of hypertension, obesity and LHV. Different allometric height-based adjustments have been used. Height^2.7^, derived from regression models in normal samples from De Simone and coworkers [26], appears to offer the most accurate estimation of LV hypertrophy and risk factors for pathologic changes in the heart structure, particularly in obese subjects. Zoccali and colleagues found LVH indexed by height^2.7 ^to be a better predictor of cardiovascular events than LVH indexed using body surface area in a group of patients under dialysis [27]. Liao and colleagues [28] studied 988 patients and identified progressive increments in death rates with both body surface area and with height^2.7 ^indexing criteria. Subjects simultaneously classified as LVH with body surface area and height^2.7 ^criteria had increased average LV mass and a 3-fold increase in death rates, while those classified as LVH only when indexed by height indexes had no increase in future cardiovascular events. In summary, it appears prudent to favor indexes that do not adjust for obesity, such as height, and height^2.7^, particularly in studies in which the independent impact of obesity is in question. Body surface area indexing permits adequate classification of most of patients in clinical practice, incorporating in LVH determination some of the risk associated with obesity.

Finally, men have increased LV mass and at least part of this effect can be attributed to body size differences. Gender differences in LV mass are first noticed around puberty and can be minimized although not eliminated by adequate indexing of body size [29]. Due to this difference in LV mass, some criteria for body size adjustments use gender-specific cut points for normality as will be seen below.

### Determining Cut-off Points

The determination of cut points in biological variables to define abnormality is frequently a source of controversy, and can be driven by different strategies. The definition of what constitutes an abnormal LV mass is no exception to this rule.

#### Left Ventricular hypertrophy diagnosis defined by deviation from mean

LV mass as most biological variables are statistically distributed in normal or skewed curves. One can consider the diagnosis of LV hypertrophy in those who are in the extreme right tail of a "Gaussian" distribution, such as beyond two standard deviations of a reference sample of normal individuals. Identification of a "normal representative sample" is not trivial and most studies use relatively small samples. In the late 80s, Levy and coworkers. [18] published a landmark paper evaluating a subset of individuals without known cardiovascular risk factors in the Framingham Cohort. These authors calculated LV mass both with the ASE convention and Troy equation (Formula 1) and with the Penn Convention and Devereux equation (Formula 2) to estimate LV mass, and proposed normal limits for LV mass for men and women, based on cut points at two standard deviations above the mean and using several indexes for body size correction (Table 2). These criteria are widely used in clinical practice and research, despite limitations in representativeness, as they may not perform well in non-white populations.

**Table 2:** Left ventricular hypertrophy cut points (Healthy reference group from The Framingham cohort).

	Men		Women	
	Mean	Mean + 2sd	Mean	Mean + 2sd

LVM(ASE) (g)	208	294	145	198
LVM(Penn) (g)	177	259	118	166
LVM/BSA(ASE) (g/m^2^)	109	150	89	120
LVM/BSA(Penn) (g/m^2^)	92	131	72	100
LVM/Ht(ASE) (g/m)	117	163	89	121
LVM/Ht(Penn) (g/m)	99	143	73	102

Adapted from[18].

#### Left Ventricular hypertrophy defined by prediction of clinical disease

Increase in LV mass has been shown to be an independent prognostic factor for intermediate endpoints [30] and clinical outcomes such as major cardiovascular events and mortality [31,32]., total mortality [27,28,33] and sudden death [34]. However, the risk associated with increases in several "physiological" variables is mostly linear over a great range of variation. This behavior has already been suggested for blood pressure [35] and cholesterol levels [36], leading to aggressive management strategies ("the lower the better"). In fact, Levy and colleagues demonstrated a progressive increase in risk associated to LV mass, even at levels not considered as "hypertrophic". Cardiovascular disease and death rates had a 1.5-fold increase for each 50 g/m of LV mass indexed by height [31]. In a subset of hypertensive patients [32], cardiovascular disease increased monotonically with more than a 4-fold increase in risk between the lowest and highest LV mass quintiles. In this study, clinically relevant increment in risk was identified in patients with LV mass below the limits usually employed for LVH definition.

These findings suggest that traditional cut-off limits may ignore cardiovascular risk associated with increased LV mass in the "normal" range based on statistical assumptions. De Simone and coworkers introduced the concept of inappropriate LV mass increase [37] to use LVH within the context of risk prediction employing multiple factors. It is considered a clinical relevant LV mass increase values above 128% of a predicted LV mass based on gender, estimated stroke volume and height^2.7 ^[38,39]

Moreover, LVH regression has been used in clinical trials as a favorable prognostic marker. A metanalysis has shown in hypertensive patients that the regression of LVH predicts a reduction of more than 50% in cardiovascular events[40]

## Evaluation of Left Ventricular Structure

Alternative concepts to LVH in the determination of left ventricular adaptive processes that take place in the overloaded ventricle assesses the fundamental components used in LV mass estimations, namely wall thickness and diastolic chamber dimension. The expected pathophysiological response of each of these components is theoretically distinct, as pressure overload leads to increased wall thickness and volume overload leads to chamber dilation. These differences cannot be assessed solely by LV mass calculations.

### Relative Wall Thickness

Parietal thickness and its relation to LV chamber size have been recognized as measures of hypertrophy for more than 30 years [41]. Relative wall thickness (RWT) is measured in clinical studies both as: 2 * posterior wall thickness divided by LV diastolic diameter or, septal wall thickness + posterior wall thickness divided by LV diastolic diameter. Even thought these measures have been used interchangeably by some investigators, septal asymmetry (IVSTD/PWTD > 1.3) was present in about 5% of Framingham subjects [42] and can lead to an underestimation of relative thickness when only posterior wall thickness is used. The reference cut point value for increased relative wall thickness derived from upper limits of normal samples is usually 0.44 [43] or 0.45 [42], irrespective of which formula is used. RWT provides information regarding LV geometry independent of other calculations [44], precluding the requirement of most corrections. Nevertheless, significant LVH can occur without major changes in RWT, particularly when simultaneous pressure and volume overload are present.

### Geometric Patterns

**Figure 2:**
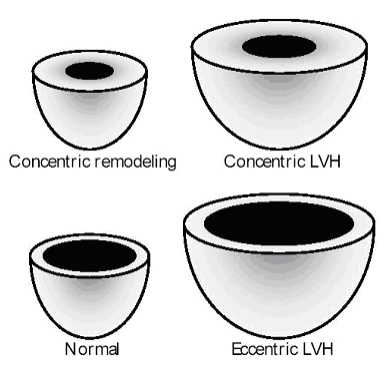
Geometric Patterns.

Attempts have been made to evaluate separately adaptive responses in parietal thickness increase and in dilation. Initially, Savage and coworkers [42] stratified Framingham patients with LVH in subgroups as: disproportionate septal LVH; concentric LVH; eccentric-dilated LVH, and eccentric non-dilated LVH. They identified in the 3 last categories increasing levels of systolic blood pressure, utilizing retrospective blood pressure data from 30-years of the cohort follow-up, suggesting a progressive character of adaptive mechanisms. A later approach defined 4 distinct geometric patterns: normal geometry, concentric remodeling, concentric hypertrophy and eccentric hypertrophy (Figure 2). Ganau and coworkers [43], using echocardiographic hemodynamic estimates, reinforced the impression that the geometric patterns parallels progressive hemodynamic changes. Hypertensive target organ disease measured by fundoscopic alterations are also more frequent in hypertrophic geometric patterns [30].

Koren and coworkers [45] used cut points of 125 g/m^2 ^for LVH and 0.45 for RWT in a sample of hypertensive patients and found a 10-year incidence of cardiovascular events of 31% in those with concentric hypertrophy compared to 11% in those with normal geometry. In 1995, two cohorts studies were simultaneously published evaluating geometric patterns impact in the incidence of cardiovascular events. Verdecchia and colleagues [46] studying 694 patients with body surface area indexed LV mass lower than 125 g/m^2^, without additional adjustment for obesity and other metabolic risk factors, found a relative risk of 2.6 in the 272 patients with concentric remodeling compared to normal geometry patients. Krumholz and coworkers [47], studied 3209 from The Framingham study, indexed LVM by height using cut points of 143 g/m in men and 102 g/m in women and adjusted the models for obesity and other relevant covariates. Their analysis showed a relative risk of 2.1 for all cause mortality with concentric hypertrophy, but not additional risk in those classified as concentric remodeling. Relative risk became nonsignificant when a correction for LV mass was included in the models. Verdecchia and colleagues [48], afterwards could not demonstrate additional risk associated with increased relative wall thickness in those classified as hypertrophic. These data may suggest a smaller independent risk associated with increased wall thickness in hypertensive patients without LVH criteria.

Even though the additional prognostic role of geometric patterns over LVH may be lesser than initially supposed, this classification permits identification of determined adaptive processes. Concentric remodeling may be related to specific pathophysiological adaptations, particularly related to glucose and insulin metabolism [49-51] and studies in contemporary cohorts have also shown an association of concentric forms with diabetes [52,53].

We believe that geometric classification, with adequate body size indexing and clearly defined standardization, may be an alternative and informative strategy to evaluate adaptive responses, providing information beyond that provided by classification with respect to left ventricular hypertrophy.

## Role of additional factors in left ventricular mass and hypertrophy determination

Gender and body size are clearly identified as predictors of LV mass and LVH Definitions are usually corrected and/or stratified for these factors, as seen above. Many others constitutional factors and exposures may lead to changes in LV mass. Some of these factors are pathophysiologically involved in LVH and, moreover, interact among themselves, limiting the interpretation of the independent role of each one.

### Gender

Differences in LV mass due to gender, independent of questions related to body size, may have pathophysiological implications. Women have been shown to have an increased parietal hypertrophic responses to pressure overload [54,55]., even after body size correction. This adaptive pattern was demonstrated also in animal models [56]. The unfavorable prognostic implications of this hypertrophic response are suggested by the findings of Liao and coworkers of a 5-fold greater risk of death associated with LV hypertrophy indexed by BSA in woman compared to the risk associated to LVH in men. However, despite using gender specific cut-offs for LVH, additional adjustment for obesity was not performed. Employment of height^2.7 ^indexing allowed to use a unique cut-point of 51 g/m^2.7 ^for both genders [26], reducing the impact of gender in LVH inference, at least in African-Americans [57].

### Obesity

Although the best strategy to adjust LVM for obesity is a matter of debate, obesity is increasingly recognized as an independent predictor of cardiovascular morbidity and mortality [58,59]. The increase in LV mass related to obesity is probably more than a mere physiologic adaptation.

Obesity has been shown to be independently associated to LVH [60], particularly in populations with a high prevalence of hypertension and other metabolic risk factors [61,62]. Despite this association, the impact of obesity on LVH may be less than expected [63], as Iacobellis and colleagues [64] have demonstrated that "uncomplicated obesity" was not a risk factor for LVH when indexed by either body surface area or height ^2.7^. As obesity, however, causes complications, it is frequently accompanied by additional risk factors. Adjusting by height ^2.7 ^minimizes the interference of obesity in LV mass estimates (See above: Body-size Indexing).

### Age

LV mass progressively increases during aging [65], particularly parietal thickness [4], which was seen in both normotensive and hypertensive patients [66]. Heart size increases during infancy and adolescence due to body size enlargement and, at this stage, the gender differences become prominent [67]. The rate of LV mass increase due to age changes in magnitude [29], weakening its independent role at older individuals, when other risk factors play a greater role [63]. Dannenbeg and coworkers [68] demonstrated that LV mass did not increase with age in a healthy sub-sample of The Framingham study, suggesting that most of the supposed physiological increase is caused by other determinants. These results are reinforced by studies in younger subjects where the age-associated increase in LV mass is partially explained by body size and blood pressure changes [67]. Nevertheless, it appears prudent to adjust for age in epidemiological investigations related to LV mass and hypertrophy.

### Ethnicity

LVH is particularly prevalent in African-Americans [5,62,69-72]. In these analyses, two particular aspects deserve consideration. An increased crude prevalence of LVH in African-Americans and Hispanics is more evident using height-indexed LV mass than with body surface area-indexed LV mass [71], suggesting that obesity may partially explain the reported ethnic differences. Furthermore, adaptive response to hypertension may differ across ethnic groups. Hypertensive African-Americans, in comparison with hypertensive whites, have increased relative wall thickness, resulting in an increased frequency of concentric remodeling, given equivalent LV mass estimates [72,73]. However, Afro-American ancestry has been identified as an independent risk factor for LVH [74].

## Clinical correlates of left ventricular hypertrophy

Several factors have been shown repeatedly in epidemiologic studies to associate with LVH. Investigation and prognostication based on LVH should take these factors in the account.

### Blood Pressure and Hypertension

Numerous population based studies have unequivocally shown an association between hypertension and LVH [4,5,65,75]. Other reports usually stratify their analysis by or restrict to those with hypertension to allow better evaluation of additional risk factors [45,73,76,77]. It is interesting that even within the normal range, increases in blood pressure is related to an increased LV mass [67]. This increment may be attributed to the classical pathophysiological concept of hypertrophic response to increased overload, although neuro-humoral and genetic factors have been also implicated [78]. LVH association with hypertension is so evident that it is recognized as target organ damage in hypertensive disease by several clinical practice guidelines, representing an intermediate unfavorable prognostic marker [79,80].

### Diabetes and The Metabolic Syndrome

Together with obesity and hypertension, diabetes has been implicated as an important determinant of left ventricular mass in most population-based studies [5,52,62,81,82]. Myocardial and systemic mechanisms, as an increased extra-cellular matrix, vascular hypertrophy and vasoconstriction [83], have been attributed to this hypertrophic response.

An adaptive response has been shown to diverse degrees of altered carbohydrate metabolism, as in Cardiovascular Health Study [82] and in The Strong Heart Study cohort, where diabetes [52], impaired glucose tolerance [84] and insulin levels[85] where associated with increased LV mass. Although associated with an increase in left ventricular mass, hyperinsulinemia [49] and insulin resistance [50] show a stronger association with concentric remodeling. Concentric hypertrophy is more pronounced in diabetes presenting with microalbuminuria [51,86]., which could imply a progressive adaptive process.

A gender difference in the left ventricular response to diabetes, with an increase in parietal thickening, rather than hypertrophy, being prominent in women has been suggested [81,85,87].

LV mass increase is also seen in individuals with other known risk factors, as in those linked to the metabolic syndrome [62,88]., where pathophysiological aspects related to this syndrome may directly affect ventricular adaptive mechanisms.

### Other Risk Factors

A multitude of other factors have been shown to be independently related to LV mass. It should be emphasized that estimates of the relative magnitude of these factors varies according to the degree of adjustment for other known risk factors in statistical modeling. Primary valvular and myocardial disease are clearly related to LV mass increase but will not be subject of our review.

Environmental exposures such as alcohol consumption [89], salt intake [90], smoking [4,89]. and increased leisure-time physical activity in men [91] have been associated to increased LV mass. Other factors such as blood lipids, pulmonary function, the heart rate and hematocrit have also been implicated but with some inconsistency among different studies [4,75,92,93]. Also, low weight at 1 year-old has been suggested as LV hypertrophy risk factor, concordant with Barker's Theory of the fetal and early life origin of chronic disease[94].

Clinical validity and impact of such factors is controversial, but it may be important to consider them as relevant potential confounders in epidemiological studies investigating the role of novel risk factors in LVH and the role of LVH in disease prediction.

## Reproducibility

Each step in LV mass measurement is a potential source of variability. In M-mode measurement, differences of approximately 5% may translate into differences in LV mass between 8% and 15% [95], which can represent about 50 g. This variability can be attributed particularly to the measurement of wall thicknesses and border layer definition [96-98]. Reproducibility is slightly better using the ASE rather than the Penn convention [98]. Additional smaller differences in left ventricular volume determinations can also be attributed to changes in body position or circulatory loading conditions [99].

Intraobserver M-mode measurements may vary about 5% between echocardiographic studies, while interobserver variability may reach 15%. Some trials retesting patients found differences of up to 30 g between tests [100,101]. When all sources of variability are taken into account, differences in the estimates are not small, since they approach a difference in LV mass values that is associated with a clinically important increased cardiovascular risk. Strategies such as core laboratory reading, strict protocols and regular training may keep this variability in an acceptable range for clinical and epidemiologic studies.

## Comparison with other Imaging Methods

Autopsy is classically employed as the gold standard in heart hypertrophy studies, because it objectively measures LV mass. However, use of reported data is complicated by the fact that macroscopic LVH definition criteria are usually more varied than those used in non-invasive testing [102-105], as well as by the fact that different studies have applied different indexing techniques for body size.

From a histological point of view, myocytes hyperplasia is uncommon in adults. Pathologic studies and animal models suggest evaluating hypertrophy suggest that myocytes keep their integrity and functionality until an increase up to 50 -70% above normal [106]. This is concordant with the Linzbach's critical level of LVH, above which cytopathological changes occur with disruption of myocardial tissue integrity and functioning [107].

LV mass can also be calculated from angiography. Although diverse formulas have been employed and validated [13], correlation with echocardiographic calculated LV mass is fair to moderate, with correlation coefficients of between 0.50 and 0.70 [108].

Radioisotopic gated myocardial perfusion imaging with ^99m^Tc-Sestamibi has been employed to estimate LV mass. Its accuracy is limited by image construction and processing variability, resulting in a limited correlation with echocardiographic LV mass [109]. However, Maruyama and coworkers [110] found a good correlation coefficient (r = 0.96) between gated ^99m^Tc-tetrofosmin myocardial perfusion and echocardiographic based LV mass estimations, using an automated quantitative software.

Newer imaging methods have been employed in LV mass determination. Computed tomography has a good correlation with necropsy findings (r = 0.97). In vivo intrareader variability was estimated to be equivalent to 19 g and intereader 28 g [111]. Magnetic resonance imaging (MRI) has emerged as a highly reproducible and accurate imaging methodology in the evaluation of LV geometry and mass [112-115]. As a result, it is of great value in evaluating distorted ventricles and its high accuracy may partially counterbalance its costs, due to the smaller samples needed. However, echocardiography costs are considerably lower in most of the countries, there is no significant radiation exposure [116], and just a few-population based studies have used these costly and less available newer imaging techniques.

Real time three-dimensional echocardiography is still experimental, but has incorporated technical advantages in image acquisition and processing. This method may permit accurate real time LV mass measurement without the caveats of geometrical assumptions. Preliminary data suggest that real time three-dimensional echocardiography is at least as accurate and reproducible as MRI calculations [117]. Contrast echo with microbubbles also permits increasing accuracy, particularly in those with inadequate acoustic windows or with distorted ventricles [118].

## Conclusion

LV mass estimation and LVH diagnosis role in cardiovascular disease management is based on epidemiological research and also on clinical grounds. Despite more than 30 years of use echocardiography-based LVH calculation and definition are still variable among ultrasound technicians and laboratories around the world, leading to inconsistency among epidemiological studies and possibly limiting its clinical application. Several technical aspects of the echocardiographic exam can generate substantial errors in LV estimations, some of them equivalent in size to those expected to result from pathophysiological processes and therapeutic strategies. Also, adequate indexing for body size seems to be a critical point in defining pathological hypertrophy. LV mass is closely related to the other known cardiovascular risk factors, that must be taken into account concomitantly. Finally, since the risk associated to LV mass appears to be progressive, without a clear threshold, additional input can be added at different baseline risks, defined by the prevalence of other known cardiovascular risk factors. The addition of multiple newer markers, however, leads to a small increment in risk stratification capacity over formulas applying only classical risk factors [119]. Despite these limitations, the role of echocardiography in LV mass determination is of great clinical value.

Considering all the aspects reviewed, use of echocardiography in clinical studies must be standardized applying already defined criteria. In delineating a study, if two-dimensional is impractical, then two-dimensional guided M-mode, using ASE criteria and Devereux modified formula, will allow estimation of LV mass with an acceptable level of accuracy. Additionally, adequate adjustment of related covariates must be undertaken. LHV/Ht^2.7^greater than 51g/m^2.7 ^appears to be a reliable criteria to define LVH, and the inclusion of a measurement of relative wall thickness, individually or classified as geometric patterns improves the identification of the adaptive mechanisms involved.

Echocardiography is widely available all over the world and major technical improvements have been achieved in the last two decades. Given careful attention with respect to the technical aspects appraised in this review, echocardiography will remain a safe, inexpensive and accurate tool for both the clinical diagnosis and epidemiologic investigation of left ventricular hypertrophy

## Competing interests

The author(s) declare that they have no competing interests.

## Authors' contributions

All authors contributed equally to this work, read and approved the final manuscript.
